# Hearing thresholds elevation and potential association with emotional problems among 1,914 children in Beijing, China

**DOI:** 10.3389/fpubh.2022.937301

**Published:** 2022-08-04

**Authors:** Huidi Xiao, Nubiya Amaerjiang, Weiwei Wang, Menglong Li, Jiawulan Zunong, Hui En, Xuelei Zhao, Cheng Wen, Yiding Yu, Lihui Huang, Yifei Hu

**Affiliations:** ^1^Department of Child, Adolescent Health and Maternal Care, School of Public Health, Capital Medical University, Beijing, China; ^2^Department of Otorhinolaryngology, Head and Neck Surgery, Beijing Friendship Hospital, Capital Medical University, Beijing, China; ^3^Department of Otolaryngology-Head and Neck Surgery, Beijing Tongren Hospital, Capital Medical University, Beijing, China; ^4^Beijing Institute of Otolaryngology, Beijing, China; ^5^Key Laboratory of Otolaryngology, Head and Neck Surgery, Ministry of Education, Beijing, China

**Keywords:** hearing screening, Strengths and Difficulties Questionnaire (SDQ), emotional problems, children, hearing loss, hearing threshold, China

## Abstract

**Objectives:**

School-aged children may experience hearing loss and emotional problems. Previous studies have shown a bidirectional relationship between hearing loss and emotional problems in the elderly population, and we aimed to analyze the association between hearing thresholds and emotional problems in school-aged children.

**Methods:**

Based on the Beijing Child Growth and Health Cohort (PROC) study, the hearing screenings were conducted in November 2019 using pure tone audiometry. A total of 1,877 parents completed the Strengths and Difficulties Questionnaire (SDQ) to assess children's emotional and behavioral status. We used generalized linear regression analysis to assess the potential association of emotional problems with hearing thresholds, based on multiple imputed datasets with a sample size of 1,914.

**Results:**

The overall pass rate of hearing screening was 91.5%. The abnormal rate of SDQ total difficulties was 55.8%. Emotional symptoms were positively associated with left ear average hearing thresholds (β = 0.24, 95%*CI*: 0.08–0.40), and right ear average hearing thresholds (β = 0.18, 95%*CI*: 0.04–0.32). Conduct problems, hyperactivity/inattention, peer problems, and prosocial behaviors had no association with the pass rate of the hearing screening. Regarding emotional symptoms, boys with many fears and who are easily scared coincided with increased right ear average hearing thresholds (β = 0.67, 95%*CI*: 0.01–1.33). Girls having many worries, frequently feeling unhappy and downhearted were positively associated with left and right ear average hearing thresholds, respectively (β = 0.96, 95%*CI*: 0.20–1.73; β = 0.72, 95%*CI*: 0.07–1.37).

**Conclusions:**

The co-occurrence of hearing problems and emotional problems of children aged 6–8 in Beijing attracts attention. It is important to address undiscovered hearing loss and emotional problems from the perspective of comorbidity driving factors.

## Introduction

Hearing loss in children is becoming an urgent public health issue. For school-aged children, newly onset hearing loss remains a matter of great concern as one of the most common developmental disorders, and its prevalence increases till adulthood due to the additions of late-onset, late identified, and acquired hearing loss (1).

The WHO estimates that approximately 34 million children worldwide live with hearing loss in 2021 (2). The National Health Interview Survey results showed that, in the US, the prevalence of moderate to severe hearing loss among children and adolescents aged 3–17 years fluctuated from 0.64% in 2009–2011 to 0.68% in 2012–2014, and to 0.58% in 2015–2017 (3). The estimated prevalence of pure-tone audiometric hearing loss among 34,618 first grade primary school students in Poland was 11% (4).

Currently, newborn hearing screening is routinely conducted in some countries (5), and research interests mainly focus on evaluation of its social benefits (6, 7). A 10-year cohort study noted that some children who passed newborn hearing screening still presented varying degrees of hearing impairment in primary school (8). Hearing loss in children is influenced by a variety of factors, while the causes of delayed hearing loss are still uncertain (9, 10). Hearing screening for school-age children is no less important than newborn hearing screening (11), with a particular role to play in the early detection of hearing loss.

Childhood hearing screening can be performed by pure tone audiometry (12), acoustic conductance testing (13), speech audiometry (14), and otoacoustic emissions (15). The American Academy of Audiology Childhood Hearing Screening Guidelines stated that pure tone audiometry is a preferred method of hearing screening for school-aged children in general, while younger or less capable children should be screened with tympanic chamber acoustic conductance or otoacoustic emissions.1 In addition, the standard for minimum hearing loss (MHL) in children varies in studies, ranging from 15 to 25 dB HL (16, 17). And 20 dB HL used as MHL in this study has been proven effective in ensuring the sensitivity of screening (18).

In the first year of primary school, a time of role adaption for children who experience significant changes in a social environment, children may present emotional and behavioral problems (19). Hearing loss seems to be negligible and indiscoverable, not only affecting the child's growth and psychological health but also having a long-term impact and even reducing the quality of life in adulthood.

Previous studies reported that children with hearing abnormalities were more likely to have emotional and behavioral problems, based on the Strengths and Difficulties Questionnaire (SDQ) assessment (20, 21). Children with emotional symptoms, conduct problems, hyperactivity/inattention, peer problems, and behavioral problems may suffer more stress. A study found that long-term stress exposure may increase the risk of hearing problems (22). Emotional status may affect the hearing threshold of children, and early hearing impairment needs to be prevented for better education performance, and cognitive and social development of the children during their growth.

Cortical hemispheric asymmetries may be present in early childhood, it means the hemispheric superiority dominates in language processing (23). With further research, the concept of asymmetry in auditory processing was verified, i.e., right ear advantage: when two different auditory inputs in the two ears, people were more likely to report better hearing in the right ear (24, 25). In addition, many studies have found that atypical hemispheric language asymmetries are associated with cognitive and psychiatric disorders among children, e.g., autism (26) and development dyslexia (27). Therefore, the issue whether emotional problems may have an impact on right and left ear hearing thresholds among children is worth exploring.

This study aimed to understand the hearing thresholds of children aged 6–8 years during their transition from kindergarten to primary school, and analyze the impact of emotional problems on hearing thresholds.

## Materials and methods

### Study design and participants

**Figure 1:**
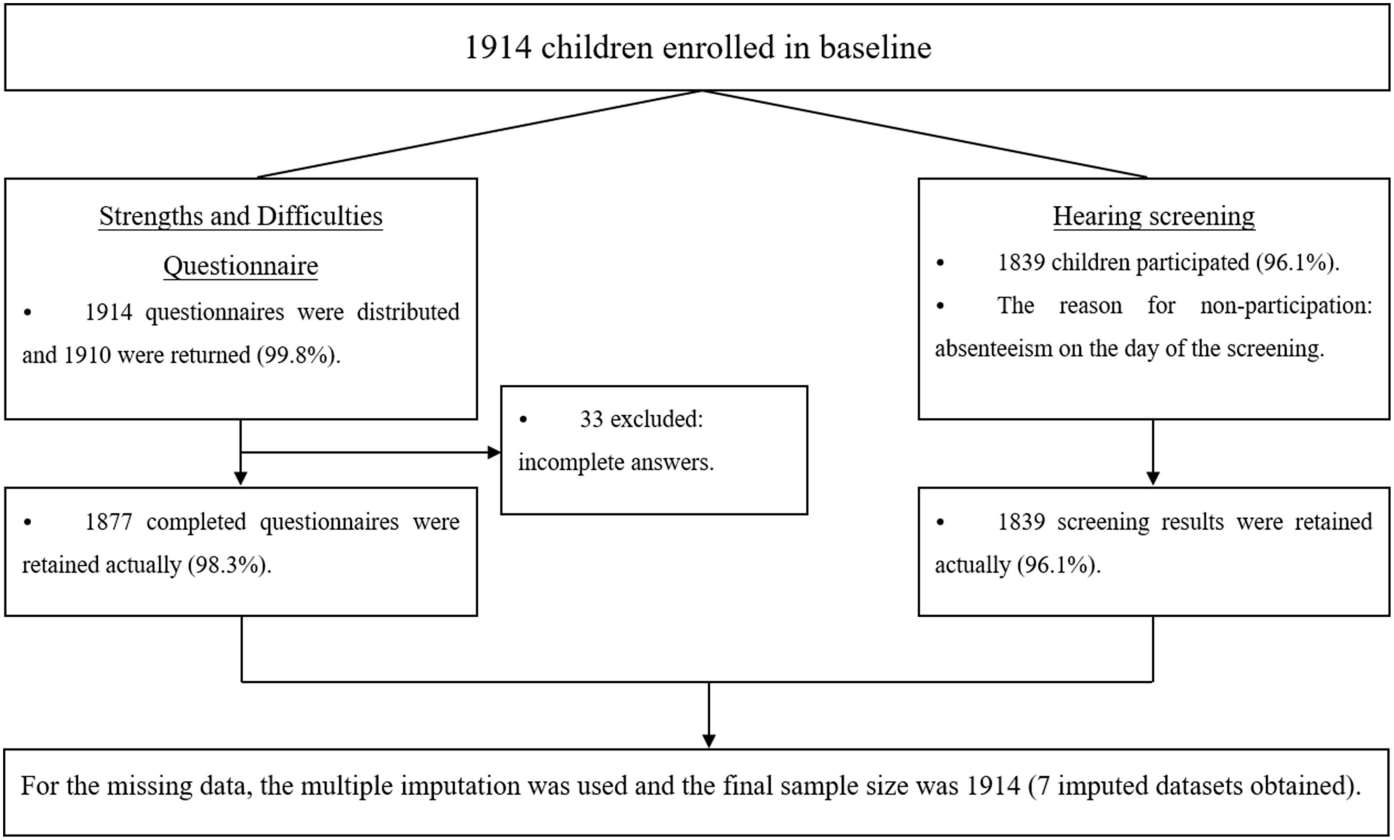
Flow chart of this study.

The participants aged 6–8 years were recruited from the Beijing Child Growth and Health Cohort (PROC) Study, launched in October 2018 in six non-boarding primary schools in Beijing (28). All parents of the children signed informed consent forms. Figure 1 showed the flow chart of this study's enrollment and exclusion process.

### Hearing screening

The hearing screenings were conducted in soundproof classrooms of each school from November 4-8, 2019. Before the screening, children were given a nasopharyngoscopy and otoscopy by an otolaryngologist to exclude possible hearing loss due to cerumen impaction and nasopharyngeal problems such as nasal obstruction. Children with cerumen impaction were included in this study after cerumen impaction was removed by the otolaryngologist. We exclude children who wore hearing aids or had otitis media because they were not suitable for hearing screening, and 1,839 of 1,914 children were screened (96.1%) except those who were absent on screening day.

Pure tone audiometry was conducted by the Madsen Xeta screening audiometer (Natus Medical Denmark ApS, Denmark) and the ambient noise was <45 dB A. The audiologists conducted the tests and explained the notes about the screening before the start of the test, to ensure that children understood the test procedure. The child was tested with right ear first (if the child reported greater hearing problems in the right ear, began with the left ear) (29) and raise his/her hand immediately upon hearing the sound from a headset, and the audiologists recorded the hearing thresholds and whether the child passed the screening.

According to the American Academy of Audiology Childhood Hearing Screening Guidelines, a pure tone sweep was conducted at 1,000, 2,000, and 4,000 Hz and 20 dB HL was used as MHL, a non-response to any frequency in either ear was considered a failure of the screening. The test sound can be given multiple times at each frequency in each ear, up to four times. For all children participating in the screening, a threshold test is performed using the “10 down, 5 up” method to determine a specific threshold as continuous measures to quantify the degree of hearing loss and those who failed were recommended for a referral to a clinical service for further diagnosis or treatment. The procedure and results of further diagnostic test were shown in the Supplementary material.

### Questionnaire

We conducted a baseline survey among the 1,914 children's parents from November 2018 to May 2019, and 1,910 parents responded (99.8%). Taking out 33 responses of incomplete answers, a total of 1,877 completed questionnaires were analyzed (98.3%).

We used the parental version of the Strengths and Difficulties Questionnaire (SDQ) to assess the emotional and behavioral status of children, including 5 scales: (a) emotional symptoms; (b) conduct problems; (c) hyperactivity/inattention; (d) peer problems; and (e) prosocial behaviors. Each scale includes 5 questions and each question has three options, “not true”, “somewhat true” and “certainly true”. The options were given different scores “0”, “1” and “2” according to the meaning of each question, and the scores were calculated in subtotal and total. We categorized scores for each scale into 3 groups of “normal”, “borderline” and “abnormal”. Each group had different cutoff values, and the instruction of the questionnaire was followed per the SDQ website (30).

### Statistical analysis

We used Statistical Analysis System V.9.4 (SAS Institute Inc., Cary, North Carolina, USA) software for statistical analysis. Multiple imputation was used to process the missing data, and 7 imputed datasets were obtained. We used means ± standard deviation to describe continuous variables for normal or proximate to a normal distribution, and used number (%) to describe categorical variables. Generalized linear regression was used to analyze the potential association of the emotional problems on the mean thresholds of the right and left ears, unadjusted and adjusted for child sex, age, BMI, and parental education (31, 32). Further analysis was conducted for statistically significant dimensions to explore the differences by sex. Two-tailed *p* < 0.05 was considered statistically significant.

## Results

Of the 1,914 children, the median age (IQR) was 6.6 (6.4, 6.9) years. Among them, 956 were boys. The boys' BMI is higher than the girls' (*p* < 0.001). The girls' maternal educational attainment is higher than the boys' (*p* = 0.018). The abnormal rate of SDQ in total difficulties reported by parents was 55.8%, with no differences between the sexes (boys was 57.4% and girls was 54.1%, *p* = 0.21). The abnormality rates were, from highest to lowest, 17.2% for peer problems, 9.3% for emotional symptoms, 9.0% for conduct problems, 8.3% for hyperactivity/inattention, and 0.0% for prosocial behaviors. The borderline rates were sequential, 16.8% for peer problems, 14.2% for prosocial behaviors, 14.0% for conduct problems, 10.2% for hyperactivity/inattention, and 10.1% for emotional symptoms. The normal rates rank as, 85.8% for prosocial behaviors, 81.5% for hyperactivity/inattention, 80.6% for emotional symptoms, 77.1% for conduct problems, and 66.0% for peer problems. Except for the conduct problems (*p* = 0.12), the reporting rates for the remaining four problems differed between sexes (*p* < 0.05). Girls presented more emotional symptoms and boys presented more hyperactivity/inattention, peer problems and prosocial behaviors (Table 1).

**Table 1:** Sociodemographic characteristics and the SDQ scores among 1,914 children aged 6–8 years in Beijing, China (imputed data).

**Characteristics**	**Total (*N =* 1,914)**	**Boys (*n =* 956)**	**Girls (*n =* 958)**	***p***
Age, $\bar{x}\;\pm \;s$	6.7 ± 0.3	6.7 ± 0.3	6.6 ± 0.3	0.24
BMI, $\bar{x}\;\pm \;s$	16.4 ± 3.0	16.9 ± 3.1	15.9 ± 2.5	<0.001
Mother's educational attainment, *n* (%)				0.018
Vocational school or below	186 (9.7)	106 (11.1)	80 (8.4)	
High school	158 (8.3)	90 (9.4)	68 (7.1)	
Junior college	599 (31.3)	308 (32.2)	291 (30.4)	
Bachelor's degree	875 (45.7)	409 (42.8)	466 (48.6)	
Master's degree or above	96 (5.0)	43 (4.5)	53 (5.5)	
Father's educational attainment, *n* (%)				0.73
Vocational school or below	241 (12.6)	129 (13.5)	112 (11.7)	
High school	180 (9.4)	93 (9.7)	87 (9.1)	
Junior college	575 (30.0)	280 (29.3)	295 (30.8)	
Bachelor's degree	826 (43.2)	410 (42.9)	416 (43.4)	
Master's degree or above	92 (4.8)	44 (4.6)	48 (5.0)	
SDQ in total difficulties SC, *n* (%)				0.21
Normal	292 (15.3)	147 (15.4)	145 (15.1)	
Borderline	555 (29.0)	260 (27.2)	295 (30.8)	
Abnormal	1,067 (55.8)	549 (57.4)	518 (54.1)	
SDQ in emotional symptoms SC, *n* (%)				0.035
Normal	1,543 (80.6)	785 (82.1)	758 (79.1)	
Borderline	194 (10.1)	99 (10.4)	95 (9.9)	
Abnormal	177 (9.3)	72 (7.5)	105 (11.0)	
SDQ in conduct problems SC, *n* (%)				0.12
Normal	1,475 (77.1)	724 (75.7)	751 (78.4)	
Borderline	267 (14.0)	149 (15.6)	118 (12.2)	
Abnormal	172 (9.0)	83 (8.7)	89 (9.3)	
SDQ in hyperactivity/inattention SC, *n* (%)				<0.001
Normal	1,559 (81.5)	746 (78.0)	813 (84.9)	
Borderline	196 (10.2)	108 (11.3)	88 (9.2)	
Abnormal	159 (8.3)	102 (10.7)	57 (5.9)	
SDQ in peer problems SC, *n* (%)				<0.001
Normal	1,263 (66.0)	595 (62.2)	668 (69.7)	
Borderline	321 (16.8)	162 (17.0)	159 (16.6)	
Abnormal	330 (17.2)	199 (20.8)	131 (13.7)	
SDQ in prosocial behaviors SC, *n* (%)				<0.001
Normal	1,642 (85.8)	792 (82.8)	850 (88.7)	
Borderline	272 (14.2)	164 (17.2)	108 (11.3)	
Abnormal	0 (0.0)	0 (0.0)	0 (0.0)	

BMI, body mass index; SDQ, Strengths and Difficulties Questionnaire; SC, score categorization.

The failure rate of the hearing screening was 8.5%, with no significant difference between the sexes. Of 162 failed children, 69.8% failed unilaterally. The hearing thresholds of the left ear at 2,000 Hz, right ear at 1,000, 2,000, and 4,000 Hz were different between sexes statistically (*p* < 0.05). And the hearing thresholds of the left ear at 4,000 Hz were marginally significant (*p* = 0.052) between sexes (Table 2).

**Table 2:** Hearing screening results among 1,914 children aged 6–8 years in Beijing, China (imputed data).

**Characteristics**	**Total (*N =* 1,914)**	**boys (*n =* 956)**	**girls (*n =* 958)**	***p***
Screening results, *n* (%)				0.88
Passing	1,752 (91.5)	876 (91.6)	876 (91.4)	
Failed	162 (8.5)	80 (8.4)	82 (8.6)	
Unilateral	113 (69.8)	53 (66.3)	60 (73.2)	
Bilateral	49 (30.2)	27 (33.7)	22 (26.8)	
**Hearing threshold**, $\bar{x}\pm \;s$				
**Left ear**				
1,000 Hz	17.1 ± 5.3	17.0 ± 5.1	17.3 ± 5.5	0.14
2,000 Hz	12.8 ± 6.5	12.4 ± 6.4	13.2 ± 6.6	<0.001
4,000 Hz	11.0 ± 7.5	10.8 ± 7.3	11.3 ± 7.6	0.052
Average	13.7 ± 5.5	13.4 ± 5.3	13.9 ± 5.7	0.004
**Right ear**				
1,000 Hz	17.0 ± 4.7	16.6 ± 4.8	17.4 ± 4.5	<0.001
2,000 Hz	12.6 ± 5.8	12.0 ± 6.2	13.2 ± 5.4	<0.001
4,000 Hz	11.2 ± 6.7	10.8 ± 7.1	11.6 ± 6.3	<0.001
Average	13.6 ± 4.8	13.1 ± 5.1	14.1 ± 4.4	<0.001
**Bilateral**				
Average	13.6 ± 4.5	13.3 ± 4.6	14.0 ± 4.3	<0.001

**Figure 2:**
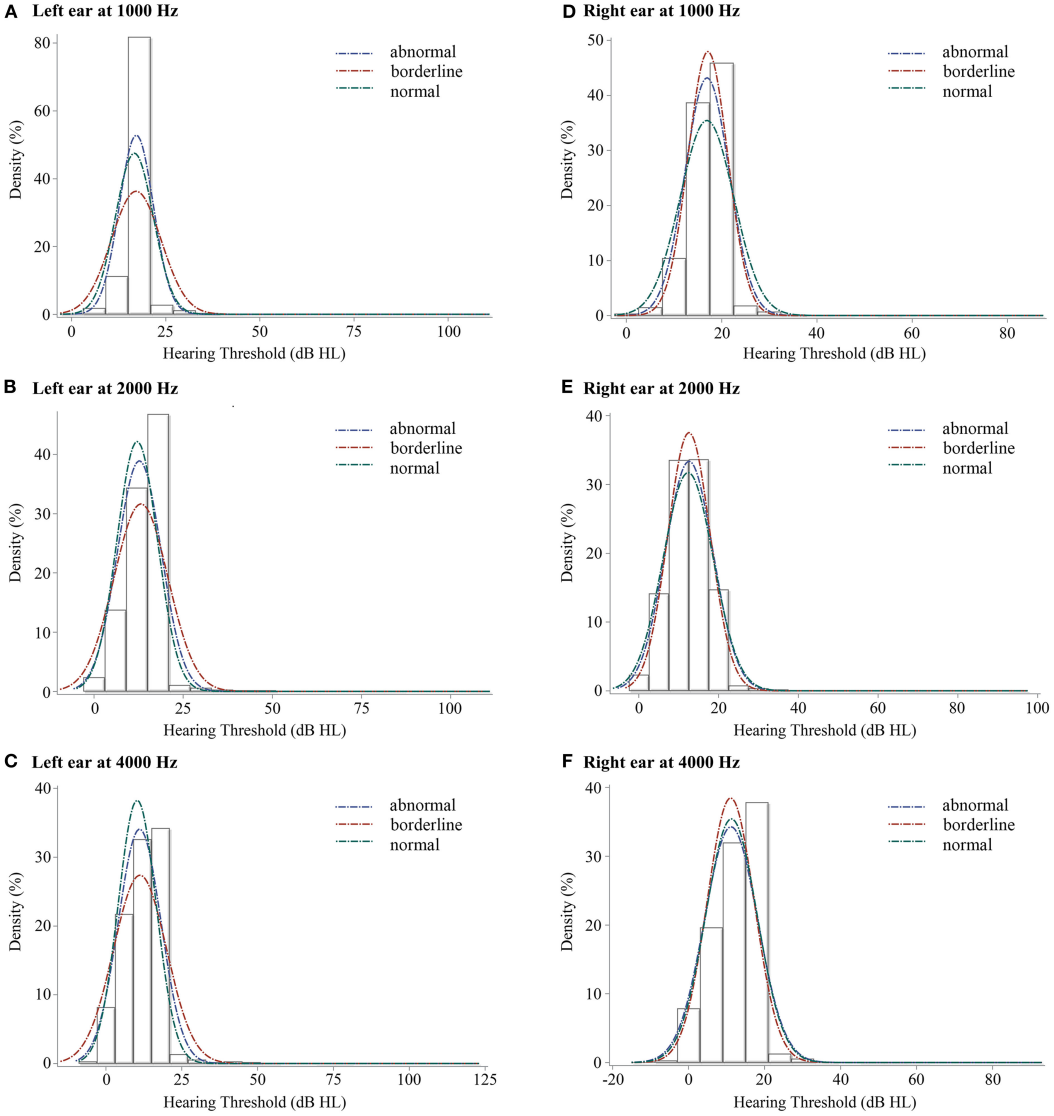
The density plots of binaural hearing thresholds at 3 frequency levels for children with SDQ total difficulties score categorization. SDQ, Strengths and Difficulties Questionnaire; **(A)**, left ear at 1,000 Hz, **(B)**, left ear at 2,000 Hz, **(C)**, left ear at 4,000 Hz, **(D)**, right ear at 1,000 Hz, **(E)**, right ear at 2,000 Hz, **(F)**, right ear at 4,000 Hz. Blue line, abnormal group of SDQ in total difficulties, red line, borderline group of SDQ in total difficulties, and green line, normal group of SDQ in total difficulties.

Figure 2 showed the hearing thresholds distribution of the 1,914 children using blue, red, and green lines reflecting the abnormal, borderline, and normal groups of SDQ in total difficulties respectively. For the left ear, the hearing threshold of the normal group was the lowest line. At 1,000 Hz, the centralized densities were sequentially ranked as abnormal, normal, and borderline groups. At 2,000 and 4,000 Hz, centralized distribution decreased in the order of normal, abnormal, and borderline groups. For the right ear at 1,000, 2,000, and 4,000 Hz, the hearing thresholds distributions of different SDQ total difficulties score categorization were similar, borderline groups top and abnormal groups follow or similar to normal groups.

Generalized linear regression showed that the emotional symptoms score was positively associated with the left ear average hearing thresholds (β = 0.24, 95%*CI*: 0.08 to 0.40, *p* = 0.004), and the right ear average hearing thresholds (β = 0.18, 95%*CI*: 0.04 to 0.32, *p* = 0.012). Adjusting sex, age, BMI of children, and parental education, the association with the left ear average hearing thresholds was similar, and the association with right ear average hearing thresholds was marginally significant (β = 0.14, 95%*CI*: 0.00 to 0.28, *p* = 0.054). Conduct problems, hyperactivity/inattention, peer problems, and prosocial behaviors did not have a significant impact on the hearing threshold (Table 3).

**Table 3:** The association of emotional problems/competence with hearing thresholds among 1,914 children aged 6–8 years in Beijing, China.

**Scales of SDQ**	**Left ear on average**				**Right ear on average**			
	***β_1_*** **(95%*CI*)**	***p***	***β_2_*** **(95%*CI*)**	***p***	***β_1_*** **(95%*CI*)**	***p***	***β_2_*** **(95%*CI*)**	***p***
Emotional symptoms	0.24 (0.08, 0.40)	0.004	0.21 (0.05, 0.38)	0.011	0.18 (0.04, 0.32)	0.012	0.14 (−0.00, 0.28)	0.054
Conduct problems	−0.08 (−0.31, 0.15)	0.49	−0.09 (−0.32, 0.14)	0.45	−0.09 (−0.29, 0.10)	0.35	−0.11 (−0.30, 0.09)	0.30
Hyperactivity/inattention	−0.07 (−0.25, 0.12)	0.47	−0.03 (−0.22, 0.16)	0.75	−0.05 (−0.22, 0.11)	0.51	−0.01 (−0.17, 0.15)	0.91
Peer problems	−0.12 (−0.30, 0.06)	0.20	−0.09 (−0.27, 0.10)	0.37	−0.10 (−0.26, 0.05)	0.19	−0.06 (−0.22, 0.09)	0.42
Prosocial behaviors	−0.00 (−0.16, 0.16)	1.00	−0.00 (−0.16, 0.16)	0.98	−0.03 (−0.16, 0.10)	0.66	−0.04 (−0.17, 0.09)	0.55

β_1_, adjusting other 4 variables of emotional problems. β_2_, adjusting sex, age, BMI of children, parental educational attainment, and other 4 variables of emotional problems.

In terms of the emotional symptoms, boys with many fears and easily scared were associated with increased right ear average hearing thresholds (β = 0.67, 95%*CI*: 0.01 to 1.33, *p* = 0.046). For girls, having many worries was positively associated with the left ear average hearing thresholds (β = 0.96, 95%*CI*: 0.20 to 1.73, *p* = 0.014) and was marginal significant on increased right ear average hearing thresholds (β = 0.57, 95%*CI*:−0.02 to 1.16, *p* = 0.060). Often being unhappy and downhearted was positively associated with the right ear average hearing thresholds (β = 0.72, 95%*CI*: 0.07 to 1.37, *p* = 0.029) (Table 4).

**Table 4:** The association of emotional symptoms with hearing thresholds among 1,914 children aged 6–8 years in Beijing, China.

**Parameters**	**Left ear on average**		**Right ear on average**	
	**β (95%*CI*)**	***p***	**β (95%*CI*)**	***p***
**Boys (*****n =*** **956)**				
Often complains of headaches	0.06 (−0.66, 0.77)	0.88	0.23 (−0.45, 0.91)	0.51
Many worries	−0.27 (−1.02, 0.48)	0.48	−0.43 (−1.14, 0.28)	0.23
Often unhappy, downhearted	−0.44 (−1.26, 0.38)	0.29	−0.43 (−1.20, 0.35)	0.28
Nervous or clingy in new situations	0.15 (−0.43, 0.73)	0.62	0.08 (−0.46, 0.63)	0.76
Many fears, easily scared	0.61 (−0.08, 1.29)	0.081	0.67 (0.01, 1.33)	0.046
**Girls (*****n =*** **958)**				
Often complains of headaches	−0.37 (−1.10, 0.35)	0.31	−0.07 (−0.64, 0.49)	0.80
Many worries	0.96 (0.20, 1.73)	0.014	0.57 (−0.02, 1.16)	0.060
Often unhappy, downhearted	0.50 (−0.34, 1.34)	0.24	0.72 (0.07, 1.37)	0.029
Nervous or clingy in new situations	0.01 (−0.62, 0.63)	0.98	−0.14 (−0.64, 0.36)	0.57
Many fears, easily scared	0.05 (−0.65, 0.75)	0.88	−0.31 (−0.85, 0.24)	0.27

Adjusting sex, age, BMI of children, and parental educational attainment.

## Discussion

We found that the hearing screening failure rate of children was 8.5% among children aged 6–8 years in Beijing, China, and the overall abnormal rate of SDQ in total difficulties score categorization reported by parents was 55.8%. The emotional symptoms were positively associated with the left and right ear average hearing threshold and presented a sex discrepancy.

The hearing screening failure rates of children varied significantly across the globe. A Poland study which also used 20 dB HL as MHL showed that the estimated prevalence of hearing loss among 5,029 children aged 6–7 years was 11.6% (33). And other two studies using 25 dB HL as the MHL presented that the prevalence of hearing impairment was 5.73% in 79,340 school-aged children in Nepal (34) and was 10% in 11,423 children in Sisimiut, Greenland (35). A 8.5% detection rate in our study seems not too high in comparison. However, given the large child population in China (253 million children aged 0–14 years in 2020 according to the seventh national census statistics), the disease burden responding to such prevalence is alarmingly high with over 21 million hearing loss cases potentially among children aged 0–14 years. Specifically, for the children who failed this screening, nearly 70% failed unilaterally. This means that a significant proportion of children have hearing loss that was slight or imperceptible, except for possible false positive results due to stress or other factors (36). However, children with unilateral hearing loss may have asymmetric speech perception and listening-related fatigue which may reduce quality of life as well (37, 38).

We found that hearing thresholds differed by sex except for the hearing thresholds of the left ear at 1,000 Hz, with higher thresholds for girls than for boys, but no sex difference in screening failure rates, which is consistent with the findings of a study on preschool children (39). This result may indicate that girls have a higher risk of hearing deterioration than boys even if they can pass the hearing screening. And a study based on the National Health and Nutrition Examination Survey (NHNES) showed that increased exposure to recreational noise and minimum use of hearing protection may contribute to the increased prevalence of noise-induced threshold shift among female youths (40). Therefore, it is important to pay attention to hearing threshold shift, especially for children with a threshold close to MHL, who need to protect their hearing and be screened regularly.

Globally, an increasing number of children and adolescents suffer from hearing loss. Due to different characteristics of hearing loss (late-onset, late identification, and late acquisition), the prevalence of hearing loss increases throughout school age (1). A meta-analysis reported an upward trend in the pooled prevalence of childhood hearing loss from 1990 to 2010 and suggested that the progress may develop slowly over decades (41). In the Recommendations for Preventive Pediatric Health Care, the American Academy of Pediatrics (AAP) emphasizes hearing screenings at each stage of childhood and adolescence (42). In addition, the American Academy of Audiology Childhood Hearing Screening Guidelines recommends that at a minimum, preschool, kindergarten, and grades 1, 3, 5, and 7 or 9 children should be screened regularly for hearing. One pure tone audiometry screening is applicable to children aged 3 years and above. Likewise in China, to protect children's hearing, to promote speech development, and also to reduce hearing and speech disabilities in children, the National Health and Family Planning Commission issued the Technical Specification for Children's Ear and Hearing Care in 2013. The technical specification recommends that all children aged 0-6 years should receive hearing screening. Screening technologies include ear appearance examination, auditory behavioral observation, and the use of portable auditory assessment instruments and otoacoustic emission (43).

This study found that the abnormal rate of SDQ total difficulties reported by parents was 55.8%, 57.4% for boys and 54.1% for girls. It is worth noting that compared to other studies, the children in this study had more severe emotional and behavioral problems. A study using the SDQ to screen for emotional and behavioral problems assessed 596 families among children aged 7–8 years and showed that the prevalence of emotional and behavioral problems was 30.0% for boys and 28.2% for girls. Hyperactivity/inattention was more prevalent among boys, which was consistent with this study (44). For children aged 6–18, the Child Behavior Checklist (CBCL) is also used to estimate emotional and behavioral problems. In this field, studies from Beijing and Nepal reported similar results, the prevalence of Total Problems were 16.7% and 18.3% respectively (45, 46). Nonetheless, a study noted that for children aged 6–18 years in Kenya, older children performed better on emotional symptoms compared to the younger (47). It was proposed that emotional problems are common in early childhood but the emotional maturity of children can be improved with age (48).

Children are only able to experience and perceive emotions when their brains develop a specific capacity (49). This is a long-term, complex and interactive process (50). This means that a child who does not have the emotional capacity cannot understand others and express him- or herself well when emotional problems arise, especially when the child has just begun school. Several studies found similar results (51–53). Children with emotional problems are at risk for elevated hearing thresholds or even hearing loss. The exact biological mechanisms are not fully understood, but a series of studies were performed to help explain the reason. Such emotional abnormalities can affect the neuroendocrine system (54). An early review suggested that there may be multiple interactions between the sympathetic and complex neuroendocrine systems, which can interact with the immune system and therefore may lead to inner ear dysfunction such as tinnitus, vertigo, and hearing loss (55). Exposure to stress hormones may cause hearing problems (56). Specifically, generalized anxiety disorder was associated with elevated inflammatory markers (57) which may contribute to the pathogenesis of hearing loss (58). Additionally, one research proposed the hypothesis: anxious personality may cause psychological distress and hearing problems (59). Although it still needs to be supported by more research, it shed light to us in future study hypothesis. That is stress or emotional problems, a matter of mental health, may have certain impact on a person's physical health or maintenance of normal functioning. These findings strengthened the evidence for the potential association of emotional problems with hearing. Moreover, several epidemiological studies from different countries found the same result of a bidirectional relationship between emotional problems or psychological stress and hearing loss in both adult and elderly populations (59–63). However, this result has not been found in children. Thus, we expanded the literature in this area.

One who pass the hearing screening does not mean that he or she has normal hearing certainly. People with higher hearing thresholds can manifest hearing impairment, communication difficulties, etc., even within the normal range (64, 65). It is of note that children with hearing impairment but normal audiograms have problems with cognitive processing (66). In this study, we found that children with abnormal or borderline SDQ total difficulties scores had higher hearing threshold at certain frequencies. For the 5 scales included in the SDQ, the presence of emotional symptoms may interplay with increased mean hearing threshold in both ears. Specifically, boys who had many fears and were easily scared, and girls who were often worried, unhappy, and downhearted, may have higher hearing threshold compared to their peers without emotional problems.

Our study has apparent strength. It is the first study to focus on the potential association of school-aged children's emotional problems with hearing using a prospective cohort study. There are also several limitations. The hearing screening and SDQ questionnaire surveys are cross-sectional at the baseline, so the cause-and-effect temporal relationships cannot be inferred definitively and in the future, we could continue to verify such association longitudinally. As the screening was performed in children aged 6–8 years in Beijing, the findings cannot be generalized to the entire childhood nor in other geographical areas. But it expanded knowledge in understanding children aged 6–8 years. The questionnaires were completed by parents based on their observations of the children for screening purposes of the prevalence of emotional and behavioral problems, not for diagnosis. Generally, SDQ is reliable and we can use it to identify children with a tendency for emotional and behavioral problems.

In conclusion, hearing loss and emotional problems of school-aged children in Beijing are cause for concern. We emphasized the necessity and importance of regular audiological monitoring in school settings and call for the attention to undiscovered hearing loss as well as emotional problems. This study is innovative in its exploration of the interplay of emotional problems with hearing status in children. It is expected that we can inform school health policy to address both challenges in the near future.

## Data availability statement

The raw data supporting the conclusions of this article will be made available by the authors, without undue reservation.

## Ethics statement

The studies involving human participants were reviewed and approved by Ethics Committee of Capital Medical University (No. 2018SY82). Written informed consent to participate in this study was provided by the participants' legal guardian/next of kin.

## Author contributions

YH is the principal investigator of the present study and conceptualized and designed the study. WW performed nasopharyngeal and otoscopy examination. LH, HE, XZ, CW, YY, HX, NA, ML, and JZ carried out the hearing screening. HX and NA conducted data analysis. HX and YH drafted the manuscript. YH, WW, LH, HE, XZ, CW, YY, HX, NA, ML, and JZ interpreted findings, edited, and revised the manuscript. All authors had final approval of the submitted and published versions and agreed to be accountable for all aspects of the work in ensuring that questions related to the accuracy or integrity of any part of the work are appropriately investigated and resolved.

## Funding

The study was supported in part by the National Natural Science Foundation of China (82073574, 82071064, and 81870730), the Beijing Natural Science Foundation (7202009) and the Capital Health Research and Development of Special (2022-2-1092). The sponsors have no role in study design, collection, analysis, and interpretation of data, the writing of the report, and in the decision to submit the paper for publication.

## Conflict of interest

The authors declare that the research was conducted in the absence of any commercial or financial relationships that could be construed as a potential conflict of interest.

## Publisher's note

All claims expressed in this article are solely those of the authors and do not necessarily represent those of their affiliated organizations, or those of the publisher, the editors and the reviewers. Any product that may be evaluated in this article, or claim that may be made by its manufacturer, is not guaranteed or endorsed by the publisher.

## References

[1] American Academy of Audiology: Childhood Hearing Screening Guidelines 2011. Available online at: https://www.cdc.gov/ncbddd/hearingloss/documents/aaa_childhood-hearing-guidelines_2011.pdf (accessed May 22, 2020).

[2] World Health Organization. World Report on Hearing. Geneva: World Health Organization (2021).

[3] Zablotsky B Black LI Maenner MJ Schieve LA Danielson ML Bitsko RH. Prevalence and trends of developmental disabilities among children in the United States: 2009-2017. Pediatrics. (2019) 144:e20190811. 10.1542/peds.2019-0811 31558576 PMC7076808

[4] Skarzyński PH Swierniak W Gos E Gocel M Skarzyński H. Organizational aspects and outcomes of a hearing screening program among first-grade children in the Mazovian Region of Poland. Lang Speech Hear Serv Sch. (2021) 52:856–67. 10.1044/2021_LSHSS-20-00083 34098724

[5] Therrell BL Jr. Padilla CD. Newborn screening in the developing countries. Curr Opin Pediatr. (2018) 30:734–9. 10.1097/MOP.0000000000000683 30124582

[6] Marinho ACA Pereira ECS Torres KKC Miranda AM Ledesma ALL. Evaluation of newborn hearing screening program. Rev Saude Publica. (2020) 54:44. 10.11606/s1518-8787.2020054001643 32374803 PMC7185987

[7] Yuan X Deng K Zhu J Xiang L Yao Y Li Q. Newborn hearing screening coverage and detection rates of hearing impairment across China from 2008-2016. BMC Pediatr. (2020) 20:360. 10.1186/s12887-020-02257-9 32731854 PMC7391493

[8] Watkin PM Baldwin M. Identifying deafness in early childhood: requirements after the newborn hearing screen. Arch Dis Child. (2011) 96:62–6. 10.1136/adc.2010.185819 21047829

[9] Lü J Huang Z Yang T Li Y Mei L Xiang M. Screening for delayed-onset hearing loss in preschool children who previously passed the newborn hearing screening. Int J Pediatr Otorhinolaryngol. (2011) 75:1045–9. 10.1016/j.ijporl.2011.05.022 21705096

[10] Jeong SW Kang MY Kim JR Kim LS. Delayed-onset hearing loss in pediatric candidates for cochlear implantation. Eur Arch Otorhinolaryngol. (2016) 273:879–87. 10.1007/s00405-015-3646-1 25956615

[11] Sliwa L Hatzopoulos S Kochanek K Piłka A Senderski A Skarzyński PH. comparison of audiometric and objective methods in hearing screening of school children. A preliminary study. Int J Pediatr Otorhinolaryngol. (2011) 75:483–8. 10.1016/j.ijporl.2010.12.024 21295353

[12] Skarzyński PH Swierniak W Gos E Pierzyńska I Walkowiak A Cywka KB. Results of hearing screening of school-age children in Bishkek, Kyrgyzstan. Prim Health Care Res Dev. (2020) 21:e18. 10.1017/S1463423620000183 32517843 PMC7303799

[13] Aithal V Aithal S Kei J Manuel A. Normative wideband acoustic immittance measurements in caucasian and aboriginal children. Am J Audiol. (2019) 28:48–61. 10.1044/2018_AJA-18-0065 30938562

[14] Ritchie BC Merklein RA. An evaluation of the efficiency of the verbal auditory screening for children (VASC). J Speech Hear Res. (1972) 15:280–6. 10.1044/jshr.1502.280 5047865

[15] Reiterer E Reider S Lackner P Fischer N Dejaco D Riechelmann H. A long-term follow-up study on otoacoustic emissions testing in paediatric patients with severe malaria in Gabon. Malar J. (2019) 18:212. 10.1186/s12936-019-2840-9 31234890 PMC6591898

[16] Lieu JEC Kenna M Anne S Davidson L. Hearing loss in children: a review. Jama. (2020) 324:2195–205. 10.1001/jama.2020.17647 33258894

[17] Mahomed-Asmail F Swanepoel de W Eikelboom RH. Hearing loss in urban South African school children (grade 1 to 3). Int J Pediatr Otorhinolaryngol. (2016) 84:27–31. 10.1016/j.ijporl.2016.02.021 27063748

[18] Dodd-Murphy J Murphy W Bess FH. Accuracy of school screenings in the identification of minimal sensorineural hearing loss. Am J Audiol. (2014) 23:365–73. 10.1044/2014_AJA-14-0014 25088976

[19] van den Bedem NP Dockrell JE van Alphen PM Rieffe C. Emotional competence mediates the relationship between communication problems and reactive externalizing problems in children with and without developmental language disorder: a longitudinal study. Int J Environ Res Public Health. (2020) 17:6008. 10.3390/ijerph17166008 32824870 PMC7459595

[20] Tsou YT Li B Eichengreen A Frijns JHM Rieffe C. Emotions in Deaf and Hard-of-Hearing and Typically Hearing Children. J Deaf Stud Deaf Educ. (2021) 26:469–82. 10.1093/deafed/enab022 34323978 PMC8448426

[21] Overgaard KR Oerbeck B Wagner K Friis S Øhre B Zeiner P. Youth with hearing loss: Emotional and behavioral problems and quality of life. Int J Pediatr Otorhinolaryngol. (2021) 145:110718. 10.1016/j.ijporl.2021.110718 33887550

[22] Canlon B Theorell T Hasson D. Associations between stress and hearing problems in humans. Hear Res. (2013) 295:9–15. 10.1016/j.heares.2012.08.015 22982334

[23] Yamazaki H Easwar V Polonenko MJ Jiwani S Wong DDE Papsin BC. Cortical hemispheric asymmetries are present at young ages and further develop into adolescence. Hum Brain Mapp. (2018) 39:941–54. 10.1002/hbm.23893 29134751 PMC6866426

[24] Prete G D'Anselmo A Brancucci A Tommasi L. Evidence of a Right Ear Advantage in the absence of auditory targets. Sci Rep. (2018) 8:15569. 10.1038/s41598-018-34086-3 30349021 PMC6197268

[25] Schmitz J Abbondanza F Paracchini S. Genome-wide association study and polygenic risk score analysis for hearing measures in children. Am J Med Genet B Neuropsychiatr Genet. (2021) 186:318–28. 10.1002/ajmg.b.32873 34476894

[26] Floris DL Barber AD Nebel MB Martinelli M Lai MC Crocetti D. Atypical lateralization of motor circuit functional connectivity in children with autism is associated with motor deficits. Mol Autism. (2016) 7:35. 10.1186/s13229-016-0096-6 27429731 PMC4946094

[27] Lizarazu M Lallier M Molinaro N Bourguignon M Paz-Alonso PM Lerma-Usabiaga G. Developmental evaluation of atypical auditory sampling in dyslexia: Functional and structural evidence. Hum Brain Mapp. (2015) 36:4986–5002. 10.1002/hbm.22986 26356682 PMC6869042

[28] Li M Shu W Zunong J Amaerjiang N Xiao H Li D. Predictors of non-alcoholic fatty liver disease in children. Pediatr Res. (2021). 10.1038/s41390-021-01754-6 34580427

[29] Missouri Department of Health Senior Services Division of Community Public Health. Guidelines for Hearing Screening in the School Setting. (2021). Available online at: https://health.mo.gov/living/families/schoolhealth/pdf/HearingScreeningGuidelines.pdf (accessed June 28, 2022).

[30] Website of the Strengths and Difficulties Questionnaire (SDQ). Available online at: https://sdqscore.org/ (accessed November 16, 2018).

[31] Hu H Tomita K Kuwahara K Yamamoto M Uehara A Kochi T. Obesity and risk of hearing loss: a prospective cohort study. Clin Nutr. (2020) 39:870–5. 10.1016/j.clnu.2019.03.020 30954364

[32] Qian ZJ Chang KW Ahmad IN Tribble MS Cheng AG. Use of diagnostic testing and intervention for sensorineural hearing loss in US children from 2008 to 2018. JAMA Otolaryngol Head Neck Surg. (2021) 147:253–60. 10.1001/jamaoto.2020.5030 33377936 PMC7774052

[33] Swierniak W Skarzynski PH Gos E Czajka N Matusiak M Hartwich P. Hearing screening among first-grade children in rural areas and small towns in Małopolskie Voivodeship, Poland. Audiol Res. (2021) 11:275–83. 10.3390/audiolres11020025 34203689 PMC8293175

[34] Maharjan M Phuyal S Shrestha M. Prevalence of hearing loss in school aged Nepalese children. Int J Pediatr Otorhinolaryngol. (2021) 143:110658. 10.1016/j.ijporl.2021.110658 33636508

[35] Jensen JS Schnohr C Skovsen CF Homøe P Jensen RG. Examination of hearing loss among school-aged children in Greenland. Int J Pediatr Otorhinolaryngol. (2021) 149:110865. 10.1016/j.ijporl.2021.110865 34385040

[36] Fitzpatrick EM Durieux-Smith A Whittingham J. Clinical practice for children with mild bilateral and unilateral hearing loss. Ear Hear. (2010) 31:392–400. 10.1097/AUD.0b013e3181cdb2b9 20054278

[37] McSweeny C Cushing SL Campos JL Papsin BC Gordon KA. Functional consequences of poor binaural hearing in development: evidence from children with unilateral hearing loss and children receiving bilateral cochlear implants. Trends Hear. (2021) 25:23312165211051215. 10.1177/23312165211051215 34661482 PMC8527588

[38] Bess FH Davis H Camarata S Hornsby BWY. Listening-related fatigue in children with unilateral hearing loss. Lang Speech Hear Serv Sch. (2020) 51:84–97. 10.1044/2019_LSHSS-OCHL-19-0017 31913803 PMC7251590

[39] Brodie KD David AP Kriss H Chan DK. Outcomes of an early childhood hearing screening program in a low-income setting. JAMA Otolaryngol Head Neck Surg. (2022). 10.1001/jamaoto.2021.4430 35175312 PMC8855310

[40] Henderson E Testa MA Hartnick C. Prevalence of noise-induced hearing-threshold shifts and hearing loss among US youths. Pediatrics. (2011) 127:e39–46. 10.1542/peds.2010-0926 21187306

[41] Wang J Sung V Carew P Burt RA Liu M Wang Y. Prevalence of childhood hearing loss and secular trends: a systematic review and meta-analysis. Acad Pediatr. (2019) 19:504–14. 10.1016/j.acap.2019.01.010 30872125

[42] Committee on Practice and Ambulatory Medicine, Bright Futures Periodicity Schedule Workgroup. 2017 recommendations for preventive pediatric health care. Pediatrics. (2017) 139:e20170254. 10.1542/peds.2017-0254 26324870

[43] National Health and Family Planning Commission of the People's Republic of China. The technical specification for children's ear and hearing care. Chin J Rural Med Pharm. (2013) 20:87–8. Available online at: https://kns.cnki.net/kcms/detail/detail.aspx?filename=XCYY201314054&dbname=cjfdtotal&dbcode=CJFD&v=MDAwNDFyQ1VSN2lmWU9SdkZ5bmxVTHZCUFM3U2Q3RzRIOUxOcTQ5QVlJUjZEZzgvemhZVTd6c09UM2lRclJjekY=

[44] Bach SL Molina ML Amaral PLD Reyes AN Jansen K Silva RAD. Emotional and behavioral problems: a school-based study in southern Brazil. Trends Psychiatry Psychother. (2019) 41:211–7. 10.1590/2237-6089-2017-0119 31390457

[45] Ma J Mahat P Brøndbo PH Handegård BH Kvernmo S Javo AC. Parent reports of children's emotional and behavioral problems in a low- and middle- income country (LMIC): an epidemiological study of Nepali schoolchildren. PLoS One. (2021) 16:e0255596. 10.1371/journal.pone.0255596 34343215 PMC8330921

[46] Yang Y Qi Y Cui Y Li B Zhang Z Zhou Y. Emotional and behavioral problems, social competence and risk factors in 6-16-year-old students in Beijing, China. PLoS One. (2019) 14:e0223970. 10.1371/journal.pone.0223970 31647827 PMC6812843

[47] Magai DN Malik JA Koot HM. Emotional and behavioral problems in children and adolescents in Central Kenya. Child Psychiatry Hum Dev. (2018) 49:659–71. 10.1007/s10578-018-0783-y 29387998 PMC6019427

[48] Forrest CL Gibson JL Halligan SL St Clair MC. A cross-lagged analysis of emotion regulation, peer problems, and emotional problems in children with and without early language difficulties: evidence from the millennium cohort study. J Speech Lang Hear Res. (2020) 63:1227–39. 10.1044/2020_JSLHR-19-00188 32315250

[49] Hoemann K Xu F Barrett LF. Emotion words, emotion concepts, and emotional development in children: a constructionist hypothesis. Dev Psychol. (2019) 55:1830–49. 10.1037/dev0000686 31464489 PMC6716622

[50] Yuill N Little S. Thinking or feeling? An exploratory study of maternal scaffolding, child mental state talk, and emotion understanding in language-impaired and typically developing school-aged children. Br J Educ Psychol. (2018) 88:261–83. 10.1111/bjep.12194 28984350

[51] Hughes N Sciberras E Goldfeld S. Family and community predictors of comorbid language, socioemotional and behavior problems at school entry. PLoS ONE. (2016) 11:e0158802. 10.1371/journal.pone.0158802 27379668 PMC4933363

[52] Sidera F Morgan G Serrat E. Understanding pretend emotions in children who are deaf and hard of hearing. J Deaf Stud Deaf Educ. (2020) 25:141–52. 10.1093/deafed/enz040 31828338

[53] de Castro BO Merk W Koops W Veerman JW Bosch JD. Emotions in social information processing and their relations with reactive and proactive aggression in referred aggressive boys. J Clin Child Adolesc Psychol. (2005) 34:105–16. 10.1207/s15374424jccp3401_10 15677285

[54] Quadt L Critchley HD Garfinkel SN. The neurobiology of interoception in health and disease. Ann N Y Acad Sci. (2018) 1428:112–28. 10.1111/nyas.13915 29974959

[55] Horner KC. The emotional ear in stress. Neurosci Biobehav Rev. (2003) 27:437–46. 10.1016/S0149-7634(03)00071-X 14505685

[56] Kraus KS Canlon B. Neuronal connectivity and interactions between the auditory and limbic systems. Effects of noise and tinnitus Hear Res. (2012) 288:34–46. 10.1016/j.heares.2012.02.009 22440225

[57] Chrousos GP. Stress and disorders of the stress system. Nat Rev Endocrinol. (2009) 5:374–81. 10.1038/nrendo.2009.106 19488073

[58] Chung SD Hung SH Lin HC Sheu JJ. Association between sudden sensorineural hearing loss and anxiety disorder: a population-based study. Eur Arch Otorhinolaryngol. (2015) 272:2673–8. 10.1007/s00405-014-3235-8 25115314

[59] Herr RM Bosch JA Theorell T Loerbroks A. Bidirectional associations between psychological distress and hearing problems: an 18-year longitudinal analysis of the British Household Panel Survey. Int J Audiol. (2018) 57:816–24. 10.1080/14992027.2018.1490034 30052099

[60] Liu W Yang C Liu L Kong G Zhang L. Bidirectional associations of vision loss, hearing loss, and dual sensory loss with depressive symptoms among the middle-aged and older adults in China. J Affect Disord. (2022) 301:225–32. 10.1016/j.jad.2022.01.066 35038482

[61] Wu C. Bidirectional association between depression and hearing loss: evidence from the china health and retirement longitudinal study. J Appl Gerontol. (2021) 7334648211042370. 10.1177/07334648211042370 34486422

[62] Cosh S Naël V Carrière I Daien V Amieva H Delcourt C. Bidirectional associations of vision and hearing loss with anxiety: prospective findings from the Three-City Study. Age Ageing. (2018) 47:582–9. 10.1093/ageing/afy062 29726887

[63] Kim SY Min C Lee CH Park B Choi HG. Bidirectional relation between depression and sudden sensorineural hearing loss: Two longitudinal follow-up studies using a national sample cohort. Sci Rep. (2020) 10:1482. 10.1038/s41598-020-58547-w 32001781 PMC6992784

[64] Parthasarathy A Hancock KE Bennett K DeGruttola V Polley DB. Bottom-up and top-down neural signatures of disordered multi-talker speech perception in adults with normal hearing. Elife. (2020) 9:e51419. 10.7554/eLife.51419 31961322 PMC6974362

[65] Kamerer AM Harris SE Kopun JG Neely ST Rasetshwane DM. Understanding self-reported hearing disability in adults with normal hearing. Ear Hear. (2022) 43:773–84. 10.1097/AUD.0000000000001161 34759207 PMC9010339

[66] Petley L Hunter LL Motlagh Zadeh L Stewart HJ Sloat NT Perdew A. Listening difficulties in children with normal audiograms: relation to hearing and cognition. Ear Hear. (2021) 42:1640–55. 10.1097/AUD.0000000000001076 34261857 PMC8545703

