# Supplementary material for: Hearing thresholds elevation and potential association with emotional problems among 1,914 children in Beijing, China
Source: Front Public Health. 2022 Aug 4;10:937301. doi: 10.3389/fpubh.2022.937301 (PMC9386347; doi:10.3389/fpubh.2022.937301)
Supplement: Supplementary file 1 [file Data_Sheet_1.docx]

***Supplementary Material***

1. **Procedure of the hearing diagnosis test**

Children who failed the hearing screening were recommended for a referral to a clinical service for further diagnosis. The hearing diagnosis test (pure tone audiometry) was conducted by an audiologist using the Madsen Xeta screening audiometer (Natus Medical Denmark ApS, Denmark) in the soundproof room. Before the testing, children were given the same examination as the initial hearing screening. The test included air conduction test and bone conduction test. At 250, 500, 1,000, 2,000, 4,000, and 8,000 Hz, the air conduction test was conducted with 25dB HL as the minimum hearing loss (MHL). If the child passed the air conduction test, his/her hearing was considered normal; if he/she had no response to any frequency in either ear with 25dB HL, the bone conduction test was further performed at 250, 500, 1,000, 2,000 and 4,000 Hz with 25dB HL as MHL. The child was tested with right ear first and raised his/her hand immediately upon hearing the sound from the headset, and the professionals recorded the hearing thresholds by using the "10 down, 5 up" method, children may be tested at 3,000 and 6,000 Hz in air conduction test if needed.

We determined the type of hearing loss based on two test results. If the child passed bone conduction test but not air conduction, and an average air-bone gap was > 10dB HL at 500, 1,000, 2,000 and 4,000Hz, it was considered as conductive hearing loss (1); if the child failed bone conduction test, and the average air-bone gap was ≤10dB HL, it was considered as sensorineural hearing loss, and the average air-bone gap was > 10dB HL, it was considered as mixed hearing loss.

Since unilateral hearing loss with normal hearing in the better ear, we classified the degree of bilateral hearing loss with an average air conduction threshold of ears at 500, 1,000, 2,000 and 4,000 Hz: slight (16-25 dB HL), mild (26-40 dB HL) and more than moderate (＞40 dB HL) (2, 3).

1. **Results of the hearing diagnosis test**

Of the 129 children who actually failed the initial hearing screening, 54 (41.9%) underwent diagnostic hearing testing after recommended referral. Of these, 45 (83.3%) had normal hearing, 3 (5.6%) had conductive hearing loss, 5 (9.3%) had sensorineural hearing loss, and 1 (1.9%) had mixed hearing loss (Table S1).

Table S1. The types of hearing loss among 9 children with diagnostic hearing testing.

| Type and degree of hearing loss | Conductive | | Sensorineural | | Mixed | |
| --- | --- | --- | --- | --- | --- | --- |
|  | n | Thresholds ^a^ | n | Thresholds ^a^ | n | Thresholds ^a^ |
| Unilateral | 3 | 22.5±7.8 | 3 | 77.1±42.3 | 1 | 33.8±8.9 |
| Bilateral | 0 | - | 2 | 42.5±32.9 | 0 | - |
| Slight | - | - | 1 | 21.9±5.6 | - | - |
| Mild | - | - | 0 | - | - | - |
| Moderate | - | - | 1 | 55.6±36.5 | - | - |

Note: ^a^ average air conduction thresholds of worse ear at 500, 1,000, 2,000 and 4,000 Hz.

1. **References**

1. Byun H, Park CJ, Oh SJ, Chung MJ, Cho BH, Cho YS. Automatic Prediction of Conductive Hearing Loss Using Video Pneumatic Otoscopy and Deep Learning Algorithm. Ear Hear (2022). doi: 10.1097/aud.0000000000001217

2. Lieu JEC, Kenna M, Anne S, Davidson L. Hearing Loss in Children: A Review. JAMA (2020) 324:2195-205. doi: 10.1001/jama.2020.17647

3. le Clercq CMP, Goedegebure A, Jaddoe VWV, Raat H, Baatenburg de Jong RJ, van der Schroeff MP. Association Between Portable Music Player Use and Hearing Loss Among Children of School Age in the Netherlands. JAMA Otolaryngol Head Neck Surg (2018) 144:668-75. doi: 10.1001/jamaoto.2018.0646
